# Identification of distinct profiles of glioblastoma through the immunocapture of extracellular vesicles from patient plasma

**DOI:** 10.1371/journal.pone.0315890

**Published:** 2025-03-19

**Authors:** Doina Ramona Manu, Rodica Bǎlaşa, Lavinia-Lorena Pruteanu, Victor Curean, Lucian Barbu-Tudoran, Georgiana-Mihaela Şerban, Rareş Chinezu, Adrian Bǎlaşa

**Affiliations:** 1 Center for Advanced Medical and Pharmaceutical Research, “George Emil Palade” University of Medicine, Pharmacy, Science and Technology of Targu Mures, Targu Mures, Romania,; 2 Department of Neurology, “George Emil Palade” University of Medicine, Pharmacy, Science and Technology of Targu Mures, Targu Mures, Romania; 3 1st Neurology Clinic, Emergency Clinical County Hospital of Targu Mures, Targu Mures, Romania; 4 Department of Chemistry and Biology, North University Center at Baia Mare, Technical University of Cluj-Napoca, Baia Mare, Romania; 5 Research Center for Functional Genomics, Biomedicine, and Translational Medicine, “Iuliu Haţieganu” University of Medicine and Pharmacy, Cluj-Napoca, Romania; 6 Doctoral School, “Iuliu Haţieganu” University of Medicine and Pharmacy, Cluj-Napoca, Romania; 7 Department of Molecular Biology and Biotechnology, Electron Microscopy Laboratory, Biology and Geology Faculty, Babes-Bolyai University, Cluj-Napoca, Romania; 8 Electron Microscopy Integrated Laboratory, National Institute for Research and Development of Isotopic and Molecular Technologies, Cluj-Napoca, Romania; 9 Doctoral School, “George Emil Palade” University of Medicine, Pharmacy, Science and Technology of Targu Mures, Targu Mures, Romania; 10 Department of Neurosurgery, “George Emil Palade” University of Medicine, Pharmacy, Science and Technology, Târgu Mureș, Romania; 11 Neurosurgery Clinic, Emergency Clinical County Hospital of Targu Mures, Targu Mures, Romania; Instituto do Cancer do Estado de Sao Paulo / University of Sao Paulo, BRAZIL

## Abstract

Glioblastoma (GBM), a primary brain tumor, exhibits intratumoral heterogeneity and dynamic spatial-temporal changes. GBM-derived extracellular vesicles (EVs), reflecting tumor characteristics, present potential as liquid-biopsy markers for early diagnosis and monitoring. This study aims to evaluate molecular signatures of plasma-derived EVs from GBM patients using a conventional flow cytometer. EVs have been isolated from glioma patients and healthy controls (HCs) plasma using density gradient ultracentrifugation (DGU). EVs were evaluated by bead-based multiplex analysis in a conventional flow cytometer. Principal component analysis (PCA), hierarchical clustering, and correlation analysis provided comprehensive insights into EV characteristics. EVs successfully isolated were visualized in transmission and scanning electron microscopy (STEM). Bead-based multiplex analysis in flow cytometer detected the level of 37 EV surface markers, including tumor-related, cancer stem cell, endothelial cell, and immune cell- specific antigens. PCA identified the EV surface markers that are most significant for differentiating the subjects, and hierarchical clustering revealed four distinct clusters based on EV surface marker levels. EV molecular signature demonstrated considerable heterogeneity across patient clusters. The presence of CD29 emerged not only as a defining factor for a cluster of patients, but also served as a marker to differentiate patients from HCs.

## 1. Introduction

Malignant brain tumors are one of the most feared pathologies due to their heterogeneous spectrum, aggressive evolution, and limited therapeutic approach. Therefore, establishing precise diagnosis criteria for these tumors is paramount to provide a targeted treatment [1,2].

In 2021, WHO updated the previous classification system of the central nervous system (CNS) malignancies by adding molecular profiling techniques to the already existing histological and immunohistochemical features and genetic alterations. GBM, currently recognized as an isocitrate dehydrogenase (IDH) wild type WHO grade 4 diffuse gliomas, is a primary brain tumor affecting adults and having one of the lowest survival rates despite the current standard of care being applied as early as possible [3–5].

GBMs are composed of tumor cells and tumor microenvironment (TME), which include immune and stromal cells, extracellular matrix (ECM), blood vessels, lymphatic vessels, cytokines, growth factors, and other non-cellular components. Intratumorally heterogeneity has been classically defined by transcriptome profiling [6]. Single-cell RNA sequencing has lately revealed the presence of various transcriptome subtypes within the same tumor [7,8]. The tumor functional heterogeneity is determined at genetic and epigenetic level, under microenvironmental pressure [9]. GBM shows a dynamic spatial and temporal heterogeneity due to reversible cellular phenotype shifts. Phenotypic changes take place *in vivo* in a complex TME defined by metabolic changes, oxygen and glucose levels, pH fluctuation, and therapy [10,11].

TME complexity is also defined by its stromal component, such as tumor associated fibroblasts (TAFs), glioma mesenchymal stem cells (GSCs), tumor endothelial cells (TECs) and pericytes. In TME network participate astrocytes, lymphocytes, microglia, and macrophages with different degrees of reactivity to modulate tumor immunosuppression [12,13]. The GBM microenvironment has been described as invasive and perivascular niches, in which the TECs are in contact with glioma stem cells (GSCs). In these areas, GSCs proliferate along the basal lamina of vessels. In fact, perivascular, hypoxic, immune, and extracellular matrix niches represent one single niche harboring GCSs, which have the ability of self-renewing and to differentiate into multiple lineages triggering GBM heterogeneity, plasticity, and progression [13,14]. In TME, in proximity to blood vessels, has been also identified a population of glioma-associated stromal cells (GASCs). This cell population has phenotypic and functional properties of GSCs and TAFs, and may facilitate angiogenesis, invasion, and tumor growth [12].

All these cells communicate not only by cell-to-cell interaction, but also through paracrine signaling exerted by soluble factors and EVs faithfully reflecting the status of their parental cells [15,16]. The bioactive cargo of EVs interferes in GBM cell- and microenvironment- mediated mechanisms to promote tumor phenotypic shifts [17–19]. EVs released from mesenchymal GSCs favor migratory potential, stemness, invasiveness, proliferation, and treatment resistance occurrence in tumor cells [20]. EVs released by tumor cells promote a pro-tumorigenic microenvironment suitable for tumor growth and dissemination, through the activation of stromal cells, extracellular matrix reprogramming and angiogenesis [21,22].

EVs originating from the same cell population may exhibit a remarkable spectrum of characteristics, which may change dynamically with the status of parental cells [23,24].

EVs originating from GBM cells can pass the blood-brain barrier (BBB) and deliver in the blood stream molecules specific to their cell of origin, reflecting the spatial and temporal tumor heterogeneity. Tumor-derived EVs may promote the dissemination of tumor cells and the occurrence of the pre-/pro-metastatic niche in target organs [25,26].

Considering all these aspects, plasma-derived EVs may serve as liquid-biopsy markers for early diagnosis and tumor monitoring [27]. Liquid biopsy has emerged as a minimally invasive technique, which is rapid, cheap, can be performed multiple times, and allow the surveillance of tumor progression by identifying the GB-derived EVs in either blood or cerebrospinal fluid [28].

In this study we aimed to evaluate molecular signatures of plasma-derived EVs from GBM patients, using a conventional flow cytometer and a multiplex bead-based technique.

## 2. Experimental procedures

**Fig 1:**
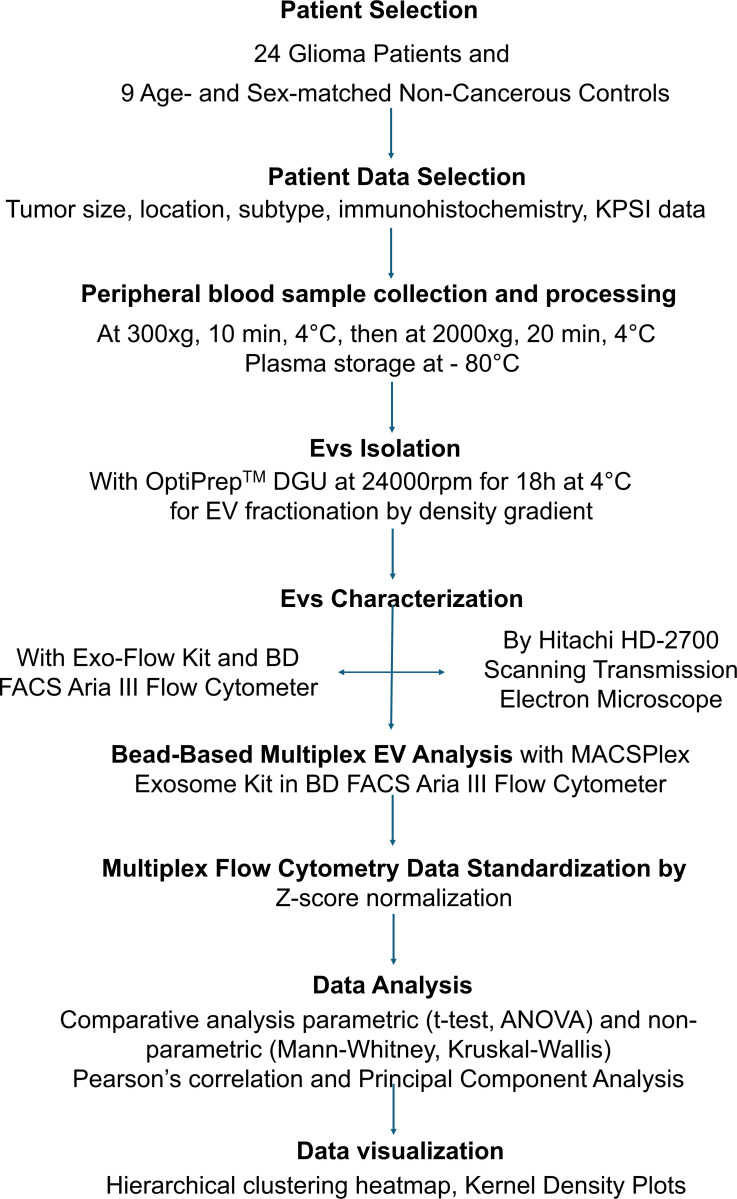
The flowchart of experimental procedures.

The workflow summarized in the Fig 1 illustrates the step-by-step experimental procedure used in this study, starting from sample preparation to data analysis.

### 2.1. Patient selection

Our study was approved by the Committee for Ethical Research of the Emergency Clinical County Hospital of Targu Mureş (decision no. 29573/8.12.2020) and the experiments were performed according to the Declaration of Helsinki principles for experiments involving humans. Patients were recruited from January 2021 until December 2022, before underwent surgical resection of the tumor. All patients signed the informed consent prior to admission to the study. Exclusion criteria were other malignancies or radio-and chemotherapy in antecedents. Peripheral blood samples were collected from 24 glioma patients and 9 healthy controls (HC) sex- and age-matched with the patients. All the patients were treated at Neurosurgery Clinic from Emergency Clinical County Hospital of Targu Mureş. Tumor size (as the largest extension in axial direction based on preoperative MRI), tumor location, molecular tumor subtype, immunohistochemical characteristics and preoperative Karnofsky Performance Status Index (KPSI) were recorded for each patient, as shown in Table 1.

**Table 1:** Main demographic, clinical and immunohistochemical characteristics of glioma patients.

Patients	Age (years)	Gender	Tumor location	Left/Right hemisphere	Tumor dimension(mm)	Preoperative KPSI	Molecular subtype	GFAP	IDH-R132H	Ki67	p53
G1.1	59	M	2	R	48	90	IDH mutant	+	+	>15%	+
G2.1	65	M	2	R	20	90	NOS	+	–	>15%	+
G6.1	68	M	0	R	43	80	IDH mutant	+	+	>15%	+
G9.1	57	M	2	R	42	90	NOS	+	–	10%	+
G10.1	74	M	2	R	71	80	NOS	+	–	8–10%	+
G13.1	55	F	1	L	29	90	NOS	+	+	20%	–
G14.1	49	M	1	L	59	80	NOS	+	–	30%	–
G15.1	67	F	3	R	37	90	NOS	+	–	25%	–
G16.1	76	F	2	L	37	80	NOS	+	–	15–20%	+
G20.1	51	F	1	R	40	60	NOS	+	–	>15%	+
G21.1	44	F	0	L	59	80	NOS	+	–	>15%	–
G23.1	67	F	0	R	34	80	NOS	+	–	35–40%	–
G25.1	79	F	2	L	40	80	NOS	+	–	10%	–
G26.1	71	M	1	R	66	80	NOS	+	–	>20%	–
G27.1	46	F	3	L	80	20	gliosarcoma	–	–	5%	–
G28.1	57	M	1	L	53	80	NOS	+	–	10%	+
G32.1	60	F	1	L	36	90	NOS	+	–	25%	+
G36.1	48	M	3	R	50	90	NOS	+	–	10%	–
G37.1	47	M	2	R	47	90	NOS	+	–	>15%	–
G38.1	62	M	0	L	54	80	NOS	+	–	10%	+
G39.1	71	M	1	L	38	90	NOS	+	–	>25%	+
G41.1	59	M	1	L	54	80	NOS	+	–	10%	–
G42.1	68	M	2	L	70	80	NOS	+	–	8–10%	–
G43.1	54	M	2	L	30	90	IDH mutant	+	+	20%	+

### 2.2. EVs isolation from GBM patient plasma

Whole blood samples were harvested from subjects in 9-mL K2-EDTA vacutainers (Becton Dickinson, Franklin Lakes, NJ, USA) and processed within 2h, according to recommendations of the current edition of the Minimal Information for Studies of Extracellular Vesicles, updated in 2023 (MISEV2023) [29]. Blood was spun at 300 × g for 10 minutes, at 4°C. Plasma samples were further centrifugated at 2000x g for 20 minutes at 4°C for platelets, cell debris and apoptotic bodies removal, then frozen at − 80°C [30]. After thawing, plasma samples were subjected to OptiPrep™ DGU method, at 24000 rpm for 18h, at 4°C, in Hitachi himac Ultracentrifuge Model CP100NX equipped with Hitachi P32ST rotor. (https://www.himac-science.com/application/life/pdf/166red_e.pdf). The density gradient was obtained with a serial dilution of 40% (1.255g/cm3), 20% (1.150g/cm^3^), 10% (1.097g/cm^3^) and 5% (1.065g/cm^3^) iodixanol (OptiPrep™ Sigma Aldrich cat. no. D1666) in Tris-HCl buffer which contained 0.25M sucrose. DGU is known as a method used to separate EV subtypes from non-vesicular extracellular particles and proteins. EVs were fractionated in the density gradient according to their specific densities. EVs comprise exosomes, microvesicles and apoptotic bodies. Knowing that the density of exosomes is between 1.15 and 1.19 g/cm^3^, the method is optimal for recovering EVs with a high purity fraction of exosomes at the border between the layers of 40% and 20% iodixanol in Tris-HCl buffer. DGU has been reported as a method leading to low recovery of high purity EVs [29]. Therefore, in this study all samples were analyzed in a single experimental run, without replication, due to limitation in sample availability. The main aim of this study was to identify early trends and gather preliminary data to offer useful insights and guide the design of future experiments, which will include replication and validation steps.

### 2.3. Evaluation of tetraspanin-positive EVs isolated by DGU

**Fig 2:**
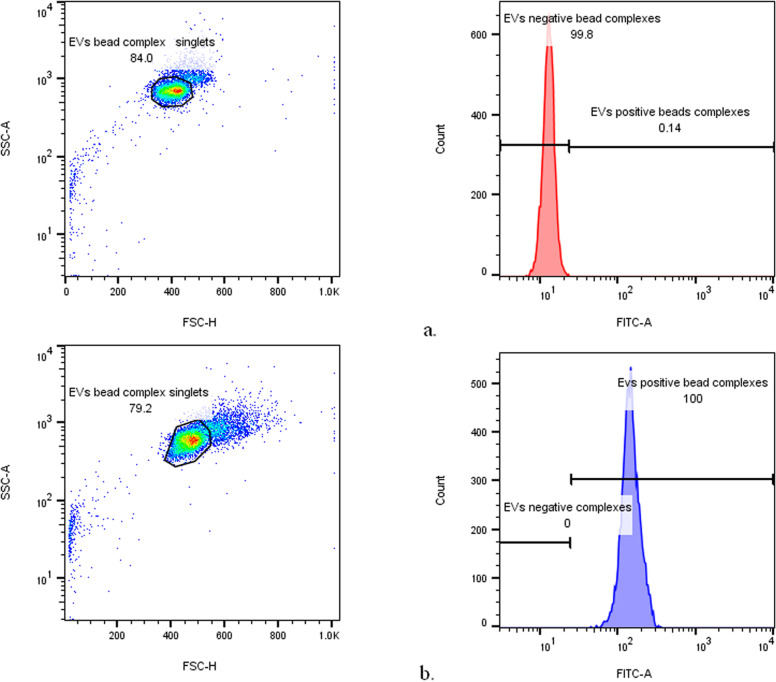
EVs positive and negative bead complexes from the control containing only buffer (a) and from a glioma patient-derived EV suspension (b).

To further assess the presence of EVs in samples collected from glioma and HC subjects using OptiPrep™ DGU, tetraspanin markers such as CD9, CD63, and CD81 were detected. Tetraspanins are proteins found on EVs originating from different cell types. This evaluation was performed with Basic Exo-Flow Capture Kit (System Biosciences cat.no. CSFLOWBASICA-1) in BD FACS Aria III Flow Cytometer. The Exo-Flow kit allows the selective capture of CD9, CD63, and CD81 positive EVs on large magnetic streptavidin beads coupled with the specific biotinylated antibodies against tetraspanins (Miltenyi biotec anti-human CD9 antibody cat.no.130-118-816, anti-human CD63 antibody cat.no. 130-100-169, anti-human CD81 antibody cat.no. 130-122-217). The captured EVs were stained with FITC conjugated to a protein that recognizes and binds EV surface proteins with post-translational modifications (glycosylations, carbohydrate additions). Tetraspanin-positive EVs determined an increase in median and mean of fluorescein isothiocyanate (FITC) fluorescence intensity during the acquisitions in the flow cytometer, as exemplified in Fig 2. The difference between the buffer control and glioma patient/HC samples is due to statistical variance of the fluorescence measurements, which increases with the number of teraspanin-enriched EVs captured on beads and subsequently stained with FITC. Plots of forward scatter (FSC) versus FITC intensity showed 0.1% of FITC-positive complexes in control (buffer only), whereas in the EVs-containing sample, over 98-99% of particles were FITC-positive in patient and HC samples. The histograms are illustrating the degree of separation between fluorescence intensity of negative versus positive complexes in the FITC channel.

### 2.4. EV characterization by Scanning and Transmission Electron Microscopy

**Fig 3:**
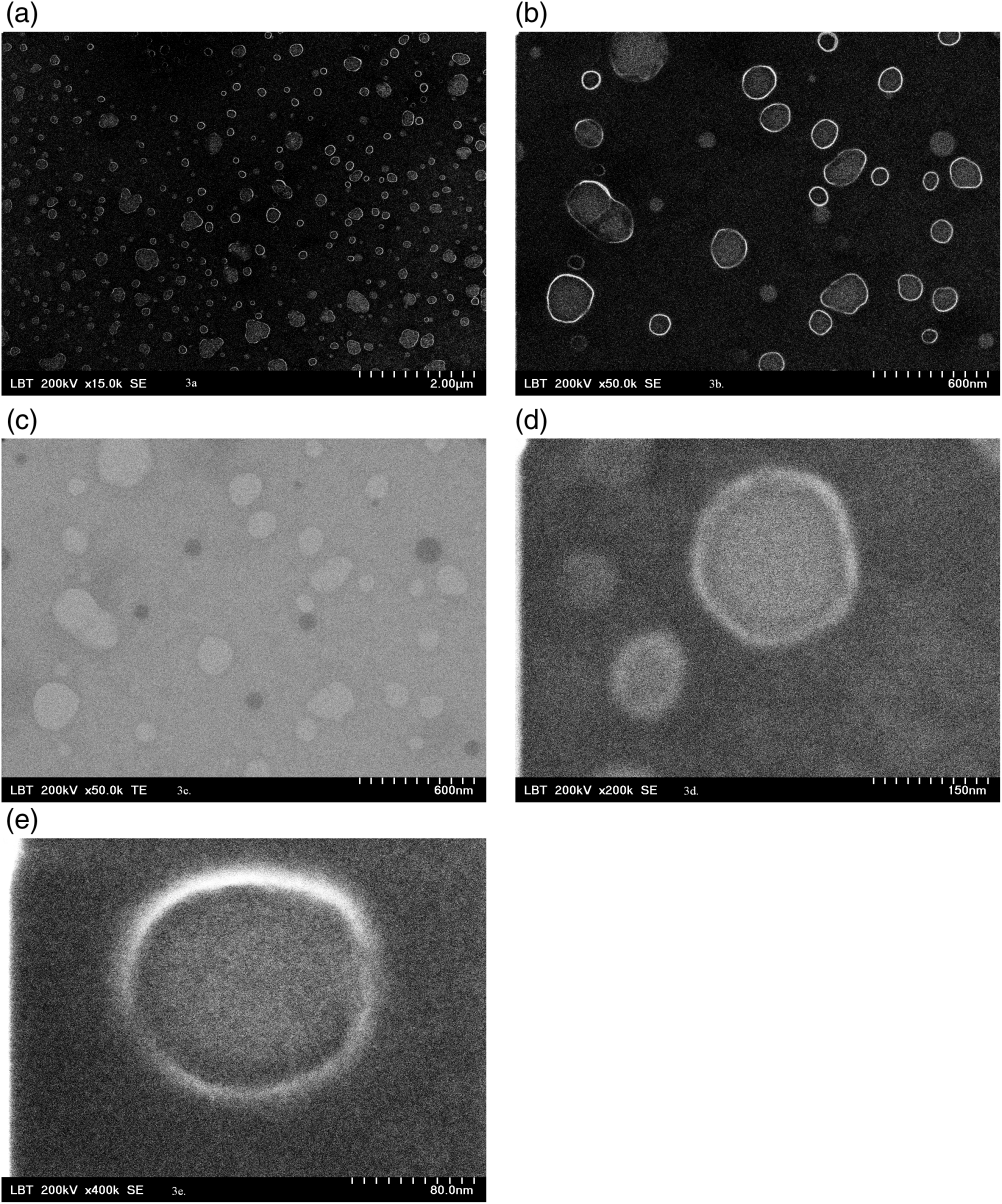
Overview of EVs suspension on SEM at magnification 15000x (a); at magnification 50000x (b); TEM at magnification 15000x (c); EVs in SEM at magnification 200000x (d); EVs in SEM at magnification 400000x (e).

As previously stated, there is no universal molecular markers of EVs or EV subtypes. TEM is among the techniques capable of detecting EVs irrespective of size [29]. EVs stored at ‒80°C were briefly thawed and mixed with an equal volume of glutaraldehyde to achieve a final concentration of 2.5%. A 5 µ L aliquot of the fixed EVs was then applied to Formvar-carbon coated grids. The EVs were visualized and imaged using a Hitachi HD-2700 scanning transmission electron microscope (Hitachi STEM High-Technologies Corp., Japan) at 200 kV, with magnifications of 15000x, 50,000x, 150000x, 200000x, 400000x, respectively, and without contrast enhancement. The nanoscale particles appeared small and rounded, typically ranging from 30 to 1000 nm, which correspond to EV sizes. The EVs exhibited an outer boundary, with variations in membrane thickness or internal content generating different contrast levels, as shown in Fig 3.

### 2.5. Bead-based multiplex EV analysis by conventional flow cytometry

EV suspensions obtained by DGU from plasma of patients and HCs were further subjected to bead-based multiplex analysis in FACS Aria III flow cytometer with MACSPlex Exosome Kit (Miltenyi Biotec, cat. no. 130-122-209).

As it was previously evaluated, this method can detect EV surface marker signatures in a specific and reproducible manner [30–32]. EV surface signatures were studied in different pathologies with the same method, using the MACSPlex Exosome Kit from Miltenyi Biotec [33–36]. The performance of the multiplex bead-based flow-cytometry assay for the detection of GBM cell EVs, primary human astrocyte-derived EVs, and circulating EVs of GBM patients was also previously evaluated [37].

In our study, a volume of 120 μL from each EVs suspension or a volume of 120μl buffer as negative control were incubated with the beads. MACSPlex Exosome Capture Beads contain 39 different antibody-coated bead subsets for the detection of 37 exosomal surface epitopes and two isotype controls. The controls including isotype-conjugated capture beads (mIgG1 and REA control) and capture beads with detection antibody alone were used according to MISEV standards [29].

15 μL of MACSPlex Exosome Capture Beads were added to EV samples (or buffer only) and incubated on an orbital shaker overnight at 450 rpm in dark, at room temperature. EVs bound by capture beads were stained with 5 μL of each APC-conjugated anti-CD9, anti-CD63, and anti-CD81 detection antibody and incubated on an orbital shaker at 450 rpm 1 h at room temperature, protected from light. Sample acquisitions were performed with BD FACSAria III cytometer and BD FACSDiva™ v 8.01 digital software. A minimum of 7500 single bead events were recorded per sample. Data were exported in FlowJo v10 software as fcs. files, and APC MFI for all 39-capture bead subset with captured EVs were background corrected by subtracting APC MFI of buffer only negative controls.

### 2.6. Statistical analysis and Data Visualization

The statistical analysis and visualization of the results was conducted with Python version 3.10.9 (Rossum, G.V. Python Programming Language. USENIX Annual Technical Conference, 2007). After the background correction by subtracting APC MFI of buffer only negative controls, the data was standardized using z-score normalization with sklearn 1.2.1. The standardized expression values were used for all subsequent analyses. For the comparison of mean expression values between GBM patients (n = 24) and HCs (n = 9), both parametric (student t-test, one-way ANOVA) and non-parametric (Kruskal-Wallis Htest, Mann-Whitney U test) tests were employed to extract significant discriminant markers between the populations using scipy 1.11.4. Differences between marker expression were statistically significant for a p-value <  0.05. Pair-wise correlations between markers were calculated with pandas 1.5.3 using Pearson’s correlation coefficient and a significant threshold of 0.05. PCA on the standardized data was performed using sklearn 1.2.1. Figures for the visualization of the results, including hierarchical clustering, correlation analysis, PCA and kernel density estimation plots were generated using seaborn 0.12.2.

## 3. Results

### 3.1. Demographic, clinical and immunohistochemical characteristics of patients

The 5th edition (2021) of WHO Classification of CNS Tumors shows that IDH-mutations are typical for diffuse lower grade astrocytomas (in the absence of 1p/19q codeletion and typically mutation in TP53 and/or ATRX) and oligodendrogliomas (with codeletion of chromosomal arms 1p and 19q). IDH-mutant gliomas have a far better prognosis than diffuse IDH wild-type gliomas. Grade I tumors are benign and slow-growing gliomas, whereas grade II tumors have the potential to develop into high-grade gliomas. High-grade gliomas can be classified as grade III and IV. Grade IV glioma or GBM has the highest malignant degree.

The study group presented in Table 1 included IDH wild-type GBM patients (n =  20), IDH-mutant astrocytoma, with mutation in isocitrate dehydrogenase 1 (IDH1) at arginine 132 (R132) (n =  3), and gliosarcoma (n =  1). Non-cancerous HC donors (n =  9) were recruited from the medical staff and patients’ relatives, without known chronic or acute conditions. The mean age of patients was 61.1 (range: 44–79) years and 54.7 (range: 41–73) years for HC. Other patient characteristics were described, such as the tumor size (ranged between 20 and 80 mm, as the largest extension in axial direction based on preoperative magnetic resonance imaging/MRI images), tumor location (frontal 0, temporal 1, parietal 2, occipital 3) and preoperative KPSI. All patients had KPSI values of 80 and 90, excepting 1 IDH wild-type GBM patient with KPSI value of 60 and gliosarcoma patient, which had the lowest KPSI value of 20 and the highest tumor size of 80 mm.

Immunohistochemical analysis showed p53 positive staining for 9 IDH wild-type GBM patients and for 3 patients with IDH-mutant astrocytoma. Negative p53 staining has been found for 12 patients from GBM group and for gliosarcoma patient. P53 is a nuclear phosphoprotein which controls the cell cycle, DNA repairing after damage and cell apoptosis. Turnover of p53 in normal cells is rapid, instead mutations in p53 gene are associated with a slower turnover and subsequent accumulation of p53 in both nucleus and cytoplasm.

In our group, the patients with > 15% positive nuclei were graded as subjects with high Ki-67 expression, and those with <  15% positive nuclei were graded as low Ki-67 expression. Only 6 patients, 5 with IDH wild-type glioblastoma and the patient with gliosarcoma were classified with a low Ki-67 expression. All the patients from our study group were reported with positive Glial fibrillar acidic protein (GFAP) staining, excepting the patient with gliosarcoma. GFAP is an intermediate filament III protein from the cytoskeleton of glia cells, associated with greater tumor invasiveness in astrocytoma. A strong GFAP positivity reflects greater cell destruction in GBM tissue and could be associated with a poor outcome.

### 3.2. The multiplex bead-based flow cytometry assay revealed the presence of immune and tumor-specific antigen expression on EV surface

Multiplex bead-based flow cytometry assay is manufactured with capture antibodies specific for exosome tetraspanin CD9, CD63, and CD81 and for surface antigens from different cell types. The beads are manufactured pre-loaded with antibodies against CD2, CD3, CD4, CD8, CD25 allowing the capture of whole plasma EVs originating from T cell subsets. The antibodies against CD56 bind EVs released by NK cell in the blood stream, the antibodies specific for CD19, CD20, CD24 bind B cell-derived EVs and anti-CD4 antibodies capture monocyte originating EVs. EVs released by the endothelial cells in the peripheral blood are captured by the antibodies against CD31 and endoglin (CD105). CD44, CD133, Epithelial cell adhesion molecule (EpCAM/CD326), stage-specific embryonic antigen-4 (SSEA-4), CD146 are expressed on the surface of EVs originating from cancer stem cells and bind their specific antibodies immobilized on the beads. Melanoma chondroitin sulfate proteoglycan (MCSP) and receptor tyrosine kinase-like orphan receptor 1 (ROR1) are known markers of tumor cells, therefore found on the tumor cell- derived EVs, which are specifically bound by antibodies from the capture beads, if these EVs are present in the suspension isolated from the subject whole plasma. EVs derived from platelets are also captured by specific antibodies against CD41b, CD42a, CD62P on the beads. Other antibodies immobilized on the beads recognize and bind HLA-ABC (MHC-I), HLA-DRDPDQ (MHC-II) and integrins (CD11c, CD29, CD41b, CD49e) -expressing EVs. On EVs released in blood stream can be detected markers of cell activation, such as CD25 (activated T, B cells and macrophages), CD69 (activated lymphocytes, monocytes and platelets), CD86 (activated lymphocytes, monocyte, dendritic cells and platelets) and CD142 (activated endothelial cells, monocytes, platelets).

Antibodies immobilized on other two bead subsets bind CD40-positive EVs (derived from T, B, dendritic cells, macrophages and endothelial cells) and CD209-positive EVs (released by dendritic cells, macrophages and endothelial cells).

**Fig 4:**
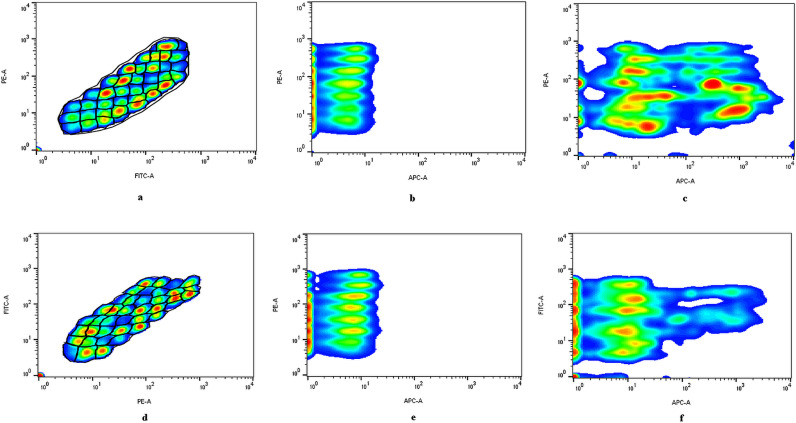
The gating strategy used for the analyze of 37 EV surface antigens from a glioblastoma patient. **a.** The 39 capture bead populations with different FITC-PE intensities; **b.** APC intensity in controls with buffer only; **c.** APC positive capture bead populations after incubation with patient-derived EV suspension. **d-f.** The gating strategy for an HC.

The gating strategy used for the analyse of 37 EV surface antigens and 2 isotypes is shown in Fig 4 a, b, c for a glioblastoma patient. 39 capture bead populations were identified in the FITC and PE channels of BD FACSAria III flow cytometer (Fig 4 a). After detection of APC positive bead populations (Fig 4 c), APC median fluorescence intensity (MFI) values for each capture bead population were background corrected by subtracting corresponding MFI values from controls (Fig 4 b). Fig 4 d, e, f shows the gating strategy for an HC subject.

Variations in APC signal intensities are semi-quantitative, as the signal originates from multiple EVs captured by individual beads. Consequently, differences in signal intensity may indicate varying EV concentrations, differences in epitope density, or distinct distributions of EV subtypes.

**Fig 5:**
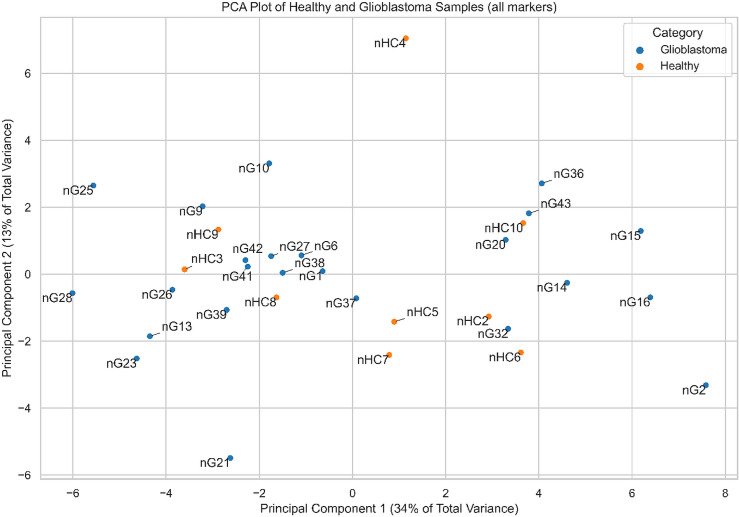
Samples of glioma patients and HCs plotted onto the first two principal components. Sample projection of the standardized expression values of all markers between glioma samples and HCs.

APC positive data recorded for each subject from the study showed a heterogeneous profile of EV surface antigens in the group. Therefore, with the APC MFI data, firstly has been created a Principal Component Analysis (PCA) to evaluate the tendency of groups based on glioblastoma conditions (+/-6 – 0), as shown in Fig 5.

### 3.3. Multiplex EV surface antigen analysis revealed four main clusters of patients showing different levels of tumor related-markers

**Fig 6:**
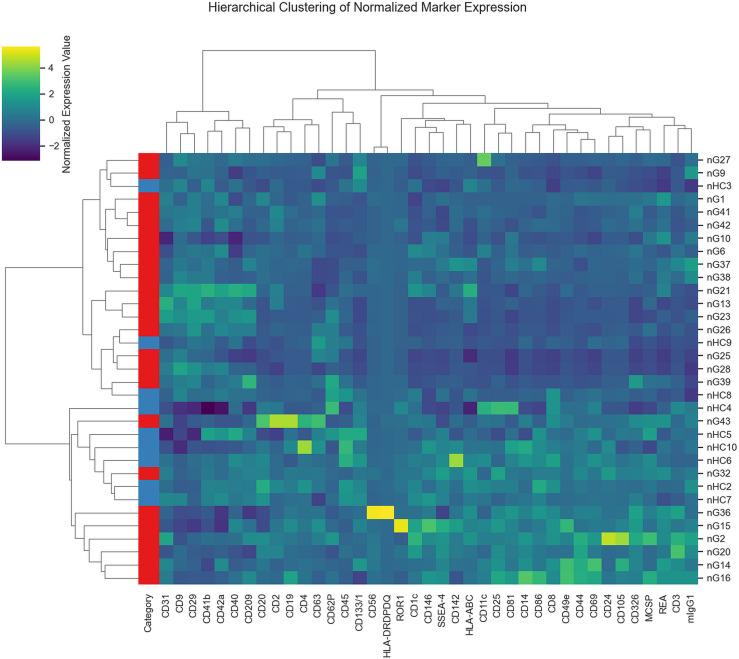
Heatmap and hierarchical clustering based on normalized marker values of glioma patients and HCs. For this plot, Ward’s minimum variance method was used for the generation of clusters with the Euclidean distance as a similarity metric.

Data analysis led to the splitting of the study group in four clusters with different levels of EV surface markers, as shown in Fig 6. The spread of the subjects in clusters is based on Pearson correlation across all markers.

The cluster composition and the presence of tumor-, stem- and endothelial cell markers on EVs, used as criteria for clustering subjects, are presented in Table 2.

**Table 2:** Surface tumor marker signatures on EVs from subjects grouped into the four clusters.

Clusters	Subject	EV Surface Marker	Key observations
1	G2	MCSP, CD326, CD44, SSEA-4, CD105, CD31	High levels of tumor cell, stem cell, and endothelial markers on EVs from peripheral blood; The highest level of CD31, CD105 positive EVs in the study group.
	G14	CD44, SSEA-4, CD105, CD31	Elevated CD44 and SSEA-4-positive EVs, suggesting the presence in the blood stream of EVs originating from stem-like cells; CD105 and CD31 positive EVs indicate high endothelial cell activity, pointing toward tumor-associated angiogenesis; this subject has a high tumor angiogenesis signature.
	G15	CD326, SSEA-4, MCSP, ROR1, CD142, CD105, CD146	The highest level of ROR1-positive EVs in the group, possibly linked to cancer metastasis and poor prognosis; the presence of EVs positives for CD142 may suggest a pro-thrombotic state, common in aggressive tumors; elevated levels of EVs positive for CD105 and CD146 in patient peripheral blood may indicate tumor endothelial dynamics.
	G16	MCSP, CD44, SSEA-4, CD146, CD105, CD31	This EV profile in patient periphery is indicative of a highly angiogenic and stem cell-enriched tumor environment.
	G20	CD44, SSEA-4	EVs with high levels of markers of stem cell-like properties, associated with tumor initiation and metastasis.
	G36	CD44, CD326	EVs with high level of CD326, related to cell adhesion, proliferation, migration, while the presence of CD44-positive EVs suggests stemness and tumor aggressiveness; this subject’s profile leans towards tumor progression rather than angiogenesis.
2	G32	CD24, CD44, SSEA-4	Higher levels of CD24, an immune evasion marker, and of CD44, SSEA-4 positive EVs compared to HCs from the cluster; CD24 is often associated with cancer cell immune evasion and metastasis; this profile reflects increased metastatic potential in contrast to HCs.
	G43	CD326, CD142	Elevated levels of CD326 and CD142- positive EVs in blood stream, compared to HCs; this profile suggests a potentially aggressive cancer phenotype.
	HC2, HC4, HC5, HC6, HC7, HC10	Low levels of tumor-defining markers on EVs	Clustered with G32 and G43 based on EV surface markers, others than tumor-defining markers.
3	G13, G21, G23, G25, G26, G28, G39	CD29, CD31	Higher levels of CD29 (integrin β1, involved in cell adhesion) and CD31-positive EVs, indicating active endothelial cell processes, aiding tumor invasion; absence of CD105-positive EVs suggests these subjects are not primarily involved in tumor angiogenesis.
	G39	CD29, CD326	High levels of EVs positive for both CD29 and CD326 in peripheral circulation of the patient; this profile emphasizes tumor progression and invasive potential.
4	G1, G6, G9, G10, G27, G37, G38	Low levels of tumor-defining markers	Similar EV marker levels as HCs; this cluster may represent non-aggressive phenotypes or possibly benign conditions.
	G41	CD29, CD31	High levels of CD29 and CD31-positive EVs, suggesting an active vascular environment with possible tumor invasion tendencies.
	G42	CD29, CD142	High levels of CD29 and CD142-positive EVs, this combination may indicate a profile prone to metastasis and clot formation.

**Fig 7:**
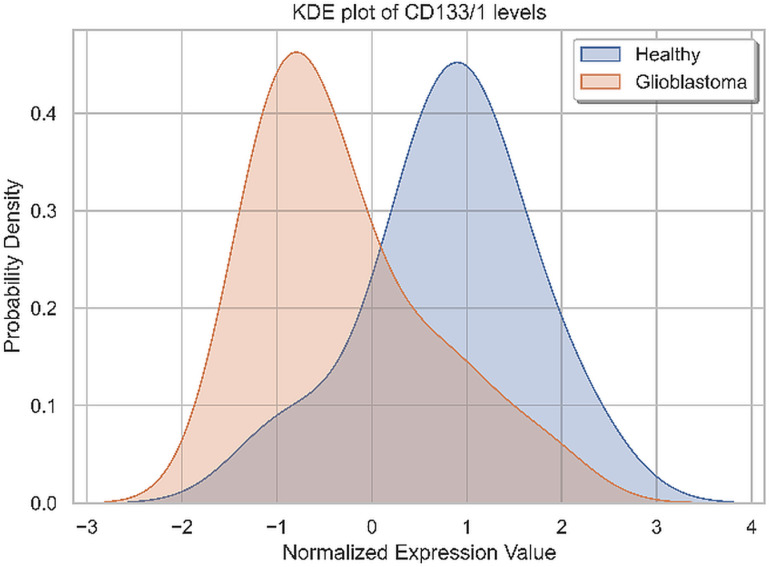
Kernel Density Estimation (KDE) plot of the CD133/1 standardized positivity in GB patients vs. HCs.

Only CD133/1, a specific cancer stem cell, could not be identified in higher levels on EVs in any patient from the group, compared to HCs. The analysis of CD133/1 in the study group is shown in Fig 7.

**Fig 8:**
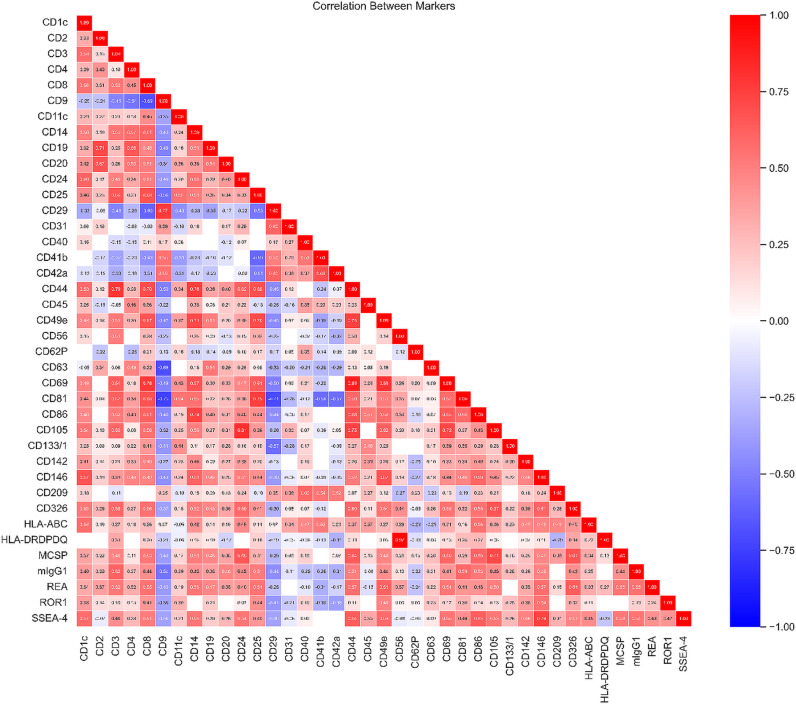
Pearson’s correlation coefficients between the standardized positivity value of the 37 markers analysed from the EV samples in the study group. A positive correlation is corresponding to a value closer to 1, while a negative correlation yields a correlation coefficient closer to -1. Statistically unsignificant correlation values are left blank.

The presence of tumor and glioma stem cell markers on EVs from patients grouped in the first two clusters is correlated with a high level of tetraspanins CD63 and CD81, as shown in Fig 8. It has been also noticed a total lack of correlation between tetraspanin CD9 and tumor or glioma stem cell marker on EVs in the subjects from these two clusters. Only EVs positives for CD31 showed high levels of tetraspanin CD9. Instead, CD105- positive EVs were positive for CD81.

The presence of tetraspanin CD9 on EVs is strongly correlated with CD29 occurrence. The third cluster of patients stands out through the presence of integrin CD29 and tetraspanin CD9 on patient-derived EVs, associated with variable levels of CD63 and CD81.

**Fig 9:**
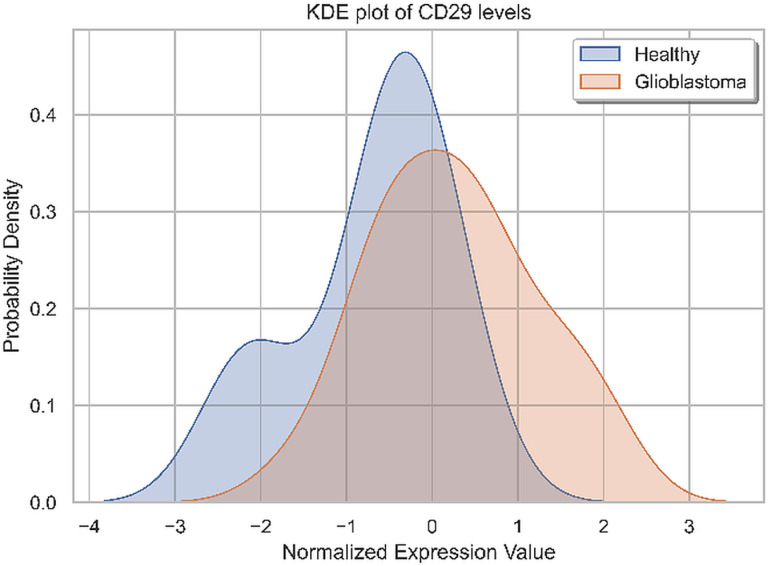
Kernel Density Estimation (KDE) plot of the CD29 standardized level in GB patients vs. HCs.

The EVs showing high levels of CD29 serve not only as defining factors in the formation of two clusters but is also a marker to distinguish the patient group from the HC group, despite the heterogeneity in CD29 expression given by lower, overlapping or higher CD29 levels compared with HC, as shown in Fig 9.

### 3.4. The heterogeneous profile of immune cell derived- EVs shows different degrees of tumor-induced immunosuppression

**Fig 10:**
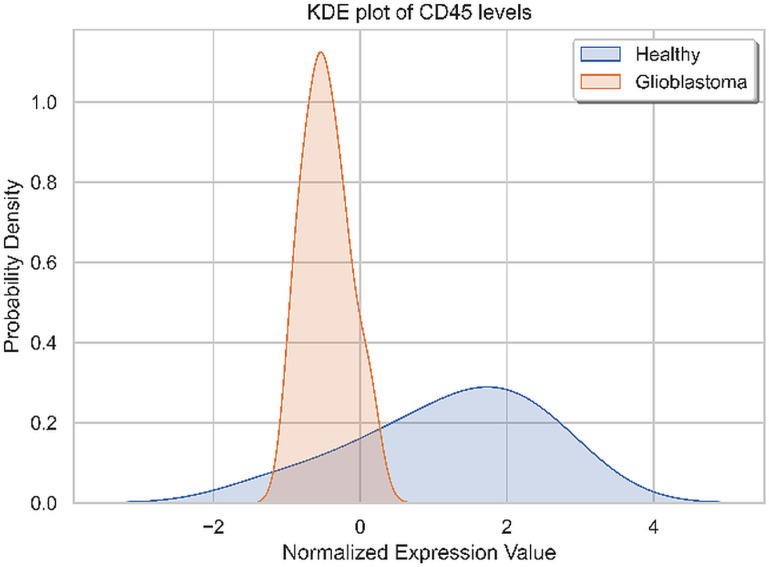
Kernel Density Estimation (KDE) plot of the CD45 standardized level in GB patients vs. HCs.

CD45 from the immune cell-derived EVs has been found as the single discriminating marker between HC group and glioma patients, with the higher presence on EVs from plasma of HCs, as shown in Fig 10.

Unlike CD45, the diverse level of antigens on the surface of EVs released by the immune cells of subjects grouped in the four clusters, added more heterogeneity to the EV profile, as described in Table 3. Only the markers of activated immune cells, such as CD86 and CD69, showed low levels on EVs isolated from plasma samples of all patients.

**Table 3:** Surface immune marker signatures on EVs from subjects grouped into the four clusters.

Clusters	EV Surface Marker	Key observations
1	Unique combination of CD3 and CD8-positive EVs, as well as CD14 and CD1c-positive EVs detected in all patients; CD25-positive EVs detected in all patients, excepting G2; G15, G36 had lower levels of EVs positives for CD14, similarly to HCs; G36 had the highest level of CD56-positive EVs from the entire study group.	High CD56 on EVs from G36 suggests NK cell anti-tumor activity.
2	G43 had the highest level of EVs positives for CD19, CD20, CD4, CD2 from the studied group; A significant amount of CD14- positive EVs together with an increased presence in plasma of EVs with CD8 and CD1c surface antigens have been also detected in this patient.	The high levels of EV in G43 may suggest strong anti-tumor immune response.
3	Cluster characterized by detection of EVs positives for CD40, CD209, CD29 and platelet-derived EVs (CD41b, CD42a, CD62P)	Presence of CD40, CD209, and platelet markers on EVs indicates a distinct immune activation profile.
4	CD11c positive EVs detected in G6, G27, G42; CD209 positive EVs present in G37, G41; CD2 positive EVs detected in G41, G42.	Cluster characterized by EV markers associated with anti-tumor immune responses (CD11c, CD209, CD2).

## 4. Discussions

The surface markers of EVs reflect both the parental cell type and its functional status. EVs share surface antigen signatures with their parent cells, making their analysis a promising tool for uncovering GBM heterogeneity, crucial for early diagnosis and prognosis.

Eguchi et al. highlighted the oncogenic potential of EVs protein cargo and introduced the conceptual term oncosome, showing that the molecular transfer of oncogenic proteins from parental to recipient cell may elicit phenotype changes and tumorigenesis [26]. The ability of EVs to cross BBB favors the transfer of information between CNS and recipient cells from periphery, leading to cellular phenotype shifts and systemic changes. The use of EVs as liquid biopsies derived from blood or cerebrospinal fluid holds great promise for glioma diagnosis, particularly for deep-seated lesions where tissue biopsies are constrained by limited accessibility and the spatial and temporal heterogeneity of the tumors.

The immunoaffinity-based EV capture from biological fluids started to be used to reveal EV molecular signatures for GBM diagnosis and prognosis. Tzaridis et al. found elevated CD81, CD44, and CD146 in serum-derived EVs from GBM patients compared to HCs. High CD29, CD44, and CD146 levels were also detected in EVs from two primary and one established high-grade glioma cell line, while CD133 was barely detectable in small EVs from all three cell lines [38]. Recently, a study performed by Franceschi et al. showed that CD105, CD133/1, CD14, CD142, CD146, CD29, CD44, CD56, HLA-DR/DP/DQ, MCSP, and the three tetraspanins CD9, CD63, and CD81 had high levels on the surface of the GBM explant-derived EVs [39]. In a study performed by Caponnetto et al., GSC-derived EVs isolated from glioma tissues after patient surgery showed high levels for CD105, SSEA-4, CD44, CD29, and CD20 and low levels for CD56, CD25, CD49e, ROR1, HLA-ABC, MCSP, and CD133 [40]. In our study, conducted on whole plasma-derived EVs, the initial PCA of the standardized dataset revealed that the variance cannot be associated with a unique GBM EV signature. EV surface antigens exhibited patient-specific signatures.The clustering process that considers the similarities or distances between patients based on EV marker profiles identified four clusters. In order to assess significant discriminant markers associated with the conditions, we conducted parametric and non-parametric statistical tests, which identified only CD29 as a tumor marker differentially expressed among glioma patients and HCs.

In the first and second clusters, patients with IDH wild-type GBM and elevated Ki-67 expression had increased levels of EV markers associated with poor prognosis, EMT transition, cell proliferation, stemness, neoangiogenesis, and therapy resistance.

The high level of MCSP-positive EVs in two GBM patients from the first cluster is consistent with previous studies showing MCSP’s association with chemo- and radioresistance, aggressive phenotype and poor prognosis in GBM [41,42]. MCSP’s functional role involves intracellular signaling pathways related to cell migration, survival, and angiogenesis, as well as interactions with membrane-type 3 matrix metalloproteinase (MT3-MMP) resulting in increased proteolysis of the ECM followed by tumor cell invasion [43]. MCSP is also a highly immunogenic tumor antigen [44].

Three patients had EVs positives for CD326/EpCAM, a glycoprotein overexpressed in glioma cells, especially in grade IV gliomas. EpCAM overexpression correlates with cell proliferation, Ki-67 expression, angiogenesis, and poor survival in high-grade gliomas [45]. EpCAM regulates various signaling pathways involved in stemness, cell proliferation, angiogenesis, EMT, metastasis, and resistance to therapy [46].

An IDH-mutant astrocytoma patient from the second cluster showed high levels of EpCAM-positive EVs associated with CD142-positive EVs, high Ki67 index, and positive p53 cells. CD142, also known as tissue factor or F3, promotes TME remodeling by activating intratumoral signaling pathways and extrinsic coagulation mechanisms, leading to mesenchymal-like cell state transition and ECM reshaping [47].

In GBM, the Ki67 proliferation index shows a positive correlation with microvessel density and CD105 and CD31 markers [48]. In the first two clusters, four patients exhibited higher levels of CD105-positive EVs compared to HCs, while only three patients displayed EV positivity for both CD31 and CD105. Patients in the third cluster, which is distinguished by EV CD29 positivity, had higher levels of CD31-positive EVs than HCs. Although GBM stem-like cells typically express endothelial markers such as CD31, CD34, and CD105, vasculogenic mimicry in GBM lacks CD31[49,50]. CD105, a transforming growth factor beta receptor upregulated in proliferating endothelial cells, is a sensitive marker for neovascularization in GBM. CD105-expressing cells in the pre-invasive niche of GBM act as a subpopulation of GBM stem-like cells, contributing to TME remodeling and GBM progression [51]. GBM cell types may release in the bloodstream EVs for CD105.

Five patients from the first cluster and one from the second cluster had higher CD44-positive EVs levels compared to HCs. CD44, a receptor for hyaluronan and osteopontin, promotes cell proliferation, cell cycle progression, and tumor-initiating cell maintenance, then tumor growth and therapy resistance [52–54]. CD44 overexpression is linked to increased glioma stemness [55]. Varying CD44 and CD133 expression in GSC types is suggesting diverse origins for GBM-derived CSCs [56]. According to Brown et al., CD133 + cells show circumscribed growth, while CD44 + cells are more invasive. CD133 expression correlates with proliferation, and CD44 with invasion. Hypoxia promotes GSC proliferation by altering CD44 and CD133 expression [57]. CD44 interacts with hyaluronic acid in the CNS, activating pathways for invasion and proliferation. In our study, CD44 + EVs were found in plasma samples, while CD133 + EVs were detected at low levels in only two patients.

In our study, five patients from the first cluster and one from the second had elevated SSEA-4 positive EV levels compared to HCs. SSEA-4, a glycosphingolipid linked to cancer cell adhesion, migration, tumor aggressiveness and resistance to chemotherapy [58,59], is associated with higher astrocytoma grades [60].

CD146 positive EVs were detected in two patients from the first cluster. CD146, a glycoprotein from the Ig superfamily, is highly expressed in GSCs from high-grade gliomas and regulates cell cycle, migration, invasion, tumor growth, and angiogenesis [61]. Higher CD146 expression is linked to tumor progression, poor survival, and treatment resistance in GBM, marking GSCs and GBM aggressiveness [62].

Only one patient had elevated levels of EVs expressing ROR1 compared to HCs. ROR1, a transmembrane protein belonging to the receptor tyrosine kinase family, is highly expressed in GBM as a stemness marker and is associated with its infiltrative nature [63]. ROR1 promotes cell proliferation, migration, and chemoresistance through various signaling pathways [64,65].

Patients in the first two clusters showed a significant presence of CD25-positive EVs, indicative of activated T regulatory lymphocytes. GBM cells release IL-6, IL-10, and express PD-L1, fostering T regulatory and myeloid-derived suppressor cell generation, impairing anti-tumor immune responses [46,66–69]. Tumor-derived EVs drive immune suppression by crossing the BBB and carrying immunomodulatory molecules between TME and the peripheral immune system.

Five out of eight patients had elevated levels of CD14-positive EVs. Immunosuppressive monocytes, known as myeloid-derived suppressor cells, characterized by CD14 expression combined with low HLA-DR were described in patients with GBM and other malignancies [70,71]. CD8-positive EVs were prevalent in six of the eight patients. The mesenchymal GBM phenotype significantly correlates with the amount of CD8 tumor- infiltrating cells, characterized by different degrees of TGF-beta-mediated suppression [72].

CD29-enriched EVs characterize the third cluster and is a discriminating marker for glioma patients. Patients with CD29-positive EVs showed endothelial CD31 positivity on EVs, but lacked CD105. CD29 (integrin β1) forms heterodimers with various α subunits (CD49a to CD49f), known as Very Late Antigens (VLA), which is involved in cell adhesion, tumor growth, invasion, angiogenesis, immune response, and therapeutic resistance. Integrins and their ligands are overexpressed in GBM cells and stromal cells within TME. Upon binding ligands from ECM, integrins activate downstream signaling pathways that promote migration, invasion, proliferation, and survival of GBM cells. Integrins signaling also triggers changes in the TME, promoting angiogenesis and immune cells trafficking [73].

EVs released by GBM cells contain β1 integrin and invasion-related proteins, contributing to tumor progression [74–76]. Proteins from β1 integrin signaling pathways were also detected in the EVs released by GBM cell lines or patient-derived GSCs [77]. In a study conducted by Spitzberg et al., multiplexed analysis of EVs showed a heterogeneous distribution of biomarkers across the EV population. The most abundant markers were CD9, CD29, CD47, CD63, CD98, CD81, and ALIX [78].

CD209, associated with myeloid cells, was upregulated in these patients, suggesting a link to immune cell infiltration in gliomas. Additionally, CD40-positive EVs were prominent, potentially linked to anti-tumor immune responses under IL-6 signaling. CD40, part of the tumor-necrosis factor receptor family, is found on various immune and tumor cells, while its ligand (CD40L) is expressed on activated T cells, platelets, and macrophages. High CD40/CD40L expression correlates with a favorable prognosis in GBM patients [79,80].

Platelets markers CD41b, CD42a, and CD62P (P-selectin) were also strongly expressed on EVs identified in patients from the third cluster. Platelets play a significant role in tumorigenesis and tumor progression, by promoting tumor angiogenesis, vascular remodeling, and stromal cell recruitment for metastatic niche formation. Conversely, tumors can activate platelets, leading to their aggregation and the release of platelet-derived factors into bloodstream. Tumors also induce thrombocytosis. Interaction between platelet P-selectin and tumor P-selectin ligand contributes to tumor growth and enhances metastasis [81,82].

The fourth cluster of glioma patients lacking classical tumor marker presence on EVs exhibit favorable clinical profiles, IDH mutations and low Ki-67 expression. Two patients harbored the IDH-R132H mutation, characteristic of lower grade astrocytomas. One patient was diagnosed with gliosarcoma, showing a low Ki-67 index. Another patient lacked the p53 mutation and achieved a postoperative KPSI of 100 at 3 months after surgery, indicating significant clinical improvement. Three patients in this cluster exhibited low Ki-67 expression. Only two patients in this cluster, who also had low Ki-67 index and were negative for p53 mutations, displayed elevated CD29-expressing EVs. One of them had both CD29 and CD142-positive EVs.

This study did not unveil a distinctive and unique EV surface antigen signature for GBM patients. Instead, it has been revealed a heterogeneous profile, with patient-specific signatures. CD29 serves as a hallmark in the third cluster, albeit with varying levels on patient EVs.

To confirm these findings, further investigation is needed, which will include replication and validation steps. The correlation between the molecular signature of tumor cells and the molecular characteristics EVs released by these cells into the bloodstream of the patients must also be investigated.

Although the study has the previously mentioned limitations, it still shows that analysis of EV surface protein signatures may provide valuable insights into the molecular and clinical diversity of GBM, highlighting potential markers for prognosis, therapeutic response, and immune modulation. The distinct EV profiles observed in different patient clusters underscore the heterogeneity of GBM and its implications for personalized treatment strategies.

## Supporting information

S1 File Raw data. (CSV)

S2 File Normalized data. (CSV)

## Abbreviations:

APC: Allophycocyanin
ATRX: Alpha thalassemia/mental retardation syndrome X-linkedBBB Blood-brain barrier
CNS: Central nervous system
DGU: Density gradient ultracentrifugation
DNA: Deoxyribonucleic acid
EpCAM: Epithelial cell adhesion molecule
ECM: Extracellular matrix
EVs: Extracellular vesicles
FITC: Fluorescein isothiocyanate
GASCs: Glioma-associated stromal cells
GBM: Glioblastoma
GFAP: Glial fibrillar acidic protein
GSCs: Glioma mesenchymal stem cells
HCs: Healthy controls
HLA: Human Leukocyte Antigen
IDH: Isocitrate dehydrogenase
KDE: Kernel Density Estimation
KPSI: Karnofsky Performance Scale Index
MCSP: Melanoma chondroitin sulfate proteoglycan
MDSC: Myeloid-derived suppressor cell
MGMT: Methylated-DNA-protein-cysteine methyltransferase
MRI: Magnetic resonance imaging
MFI: Median fluorescence intensity MFI
MT3: MMP membrane-type 3 matrix metalloproteinase
PCA: Principal Component Analysis
PD-(L)1: Programmed Cell Death Ligand 1
RNA: Ribonucleic acid
ROR1: Receptor tyrosine kinase-like orphan receptor 1
SSEA-4: Stage-specific embryonic antigen-4
TAFs: Tumor associated fibroblasts
TEC: Tumor endothelial cell
TGF-β: Transforming growth factor beta
TME: Tumor microenvironment
VE: vascular endothelial
VEGFR: vascular endothelial growing factor receptor
VLA: Very Late Antigen
vWF: von Willebrand factor.

## References

[1] Blakstad H, Brekke J, Rahman MA, Arnesen VS, Miletic H, Brandal P, et al. Survival in a consecutive series of 467 glioblastoma patients: association with prognostic factors and treatment at recurrence at two independent institutions. PLoS One. 2023;18(2):e0281166. doi: 10.1371/journal.pone.0281166 36730349 PMC9894455

[2] Pruteanu L-L, Kopanitsa L, Módos D, Kletnieks E, Samarova E, Bender A, et al. Transcriptomics predicts compound synergy in drug and natural product treated glioblastoma cells. PLoS One. 2020;15(9):e0239551. doi: 10.1371/journal.pone.0239551 32946518 PMC7500592

[3] Guan X, Vengoechea J, Zheng S, Sloan AE, Chen Y, Brat DJ, et al. Molecular subtypes of glioblastoma are relevant to lower grade glioma. PLoS One. 2014;9(3):e91216. doi: 10.1371/journal.pone.0091216 24614622 PMC3948818

[4] Le Rhun E, Preusser M, Roth P, Reardon DA, van den Bent M, Wen P, et al. Molecular targeted therapy of glioblastoma. Cancer Treat Rev. 2019;80:101896. doi: 10.1016/j.ctrv.2019.101896 31541850

[5] Louis DN, Perry A, Wesseling P, Brat DJ, Cree IA, Figarella-Branger D, et al. The 2021 WHO classification of tumors of the central nervous system: a summary. Neuro Oncol. 2021;23(8):1231–51. doi: 10.1093/neuonc/noab106 34185076 PMC8328013

[6] Isachesku E, Braicu C, Pirlog R, Kocijancic A, Busuioc C, Pruteanu L-L, et al. The role of non-coding RNAs in epigenetic dysregulation in glioblastoma development. Int J Mol Sci. 2023;24(22):16320. doi: 10.3390/ijms242216320 38003512 PMC10671451

[7] Couturier CP, Ayyadhury S, Le PU, Nadaf J, Monlong J, Riva G, et al. Single-cell RNA-seq reveals that glioblastoma recapitulates a normal neurodevelopmental hierarchy. Nat Commun. 2020;11(1):3406. doi: 10.1038/s41467-020-17186-5 32641768 PMC7343844

[8] Johnson E, Dickerson KL, Connolly ID, Hayden Gephart M. Single-cell RNA-sequencing in glioma. Curr Oncol Rep. 2018;20(5):42. doi: 10.1007/s11912-018-0673-2 29637300 PMC8403493

[9] Barthel L, Hadamitzky M, Dammann P, Schedlowski M, Sure U, Thakur BK, et al. Glioma: molecular signature and crossroads with tumor microenvironment. Cancer Metastasis Rev. 2022;41(1):53–75. doi: 10.1007/s10555-021-09997-9 34687436 PMC8924130

[10] Broekman ML, Maas SLN, Abels ER, Mempel TR, Krichevsky AM, Breakefield XO. Multidimensional communication in the microenvirons of glioblastoma. Nat Rev Neurol. 2018;14(8):482–95. doi: 10.1038/s41582-018-0025-8 29985475 PMC6425928

[11] Dirkse A, Golebiewska A, Buder T, Nazarov PV, Muller A, Poovathingal S, et al. Stem cell-associated heterogeneity in Glioblastoma results from intrinsic tumor plasticity shaped by the microenvironment. Nat Commun. 2019;10(1):1787. doi: 10.1038/s41467-019-09853-z 30992437 PMC6467886

[12] Clavreul A, Menei P. Mesenchymal stromal-like cells in the glioma microenvironment: what are these cells?. Cancers (Basel). 2020;12(9):2628. doi: 10.3390/cancers12092628 32942567 PMC7565954

[13] Schiffer D, Annovazzi L, Casalone C, Corona C, Mellai M. Glioblastoma: microenvironment and niche concept. Cancers. 2018;11(1):5. 30577488 10.3390/cancers11010005 PMC6357107

[14] Nasrolahi A, Azizidoost S, Radoszkiewicz K, Najafi S, Ghaedrahmati F, Anbiyaee O, et al. Signaling pathways governing glioma cancer stem cells behavior. Cell Signal. 2023;101:110493. doi: 10.1016/j.cellsig.2022.110493 36228964

[15] Jin Y, Xing J, Xu K, Liu D, Zhuo Y. Exosomes in the tumor microenvironment: promoting cancer progression. Front Immunol. 2022;13:1025218. doi: 10.3389/fimmu.2022.1025218 36275738 PMC9584056

[16] Zhao Y, Shen M, Wu L, Yang H, Yao Y, Yang Q, et al. Stromal cells in the tumor microenvironment: accomplices of tumor progression?. Cell Death Dis. 2023;14(9):587. doi: 10.1038/s41419-023-06110-6 37666813 PMC10477351

[17] Bălașa A, Șerban G, Chinezu R, Hurghiș C, Tămaș F, Manu D. The involvement of exosomes in glioblastoma development, diagnosis, prognosis, and treatment. Brain Sci. 2020;10(8):553. 32823792 10.3390/brainsci10080553 PMC7463943

[18] Yang E, Wang X, Gong Z, Yu M, Wu H, Zhang D. Exosome-mediated metabolic reprogramming: the emerging role in tumor microenvironment remodeling and its influence on cancer progression. Signal Transduct Target Ther. 2020;5(1):242. doi: 10.1038/s41392-020-00359-5 33077737 PMC7572387

[19] Lopez K, Lai S, Lopez Gonzalez E, Dávila R, Shuck S. Extracellular vesicles: a dive into their role in the tumor microenvironment and cancer progression. Front Cel Develop Biol. 2023;11:1154576. doi: 10.3389/fcdbi.2023.1154576 PMC10071009 37025182

[20] Schweiger MW, Li M, Giovanazzi A, Fleming RL, Tabet EI, Nakano I, et al. Extracellular vesicles induce mesenchymal transition and therapeutic resistance in glioblastomas through NF-κB/STAT3 signaling. Adv Biosyst. 2020;4(12):e1900312. doi: 10.1002/adbi.201900312 32519463 PMC7718424

[21] Li I, Nabet BY. Exosomes in the tumor microenvironment as mediators of cancer therapy resistance. Mol Cancer. 2019;18(1):32. doi: 10.1186/s12943-019-0975-5 30823926 PMC6397467

[22] Sheta M, Taha EA, Lu Y, Eguchi T. Extracellular vesicles: new classification and tumor immunosuppression. Biology (Basel). 2023;12(1):110. doi: 10.3390/biology12010110 36671802 PMC9856004

[23] Choi D, Montermini L, Kim D-K, Meehan B, Roth FP, Rak J. The impact of oncogenic EGFRvIII on the proteome of extracellular vesicles released from glioblastoma cells. Mol Cell Proteomics. 2018;17(10):1948–64. doi: 10.1074/mcp.RA118.000644 30006486 PMC6166673

[24] Schweer D, Anand N, Anderson A, McCorkle J, Neupane K, Nail A. Human macrophage-engineered vesicles for utilization in ovarian cancer treatment. Front Oncol. 2023;12:1042730. 36713536 10.3389/fonc.2022.1042730 PMC9875020

[25] Balasa R, Barcutean L, Mosora O, Manu D. Reviewing the significance of blood–brain barrier disruption in multiple sclerosis pathology and treatment. Int J Mol Sci. 2021;22(16):8370. doi: 10.3390/ijms22168370 34445097 PMC8395058

[26] Eguchi T, Sheta M, Fujii M, Calderwood SK. Cancer extracellular vesicles, tumoroid models, and tumor microenvironment. Semin Cancer Biol. 2022 Nov;86:112–26. 35032650 10.1016/j.semcancer.2022.01.003

[27] Whitehead C, Kaye A, Drummond K, Widodo S, Mantamadiotis T, Vella L. Extracellular vesicles and their role in glioblastoma. Crit Rev Clinic Lab Sci. 2020;57(4):227–52. 10.1080/10408363.2019.1700208 31865806

[28] Klekner Á, Szivos L, Virga J, Árkosy P, Bognár L, Birkó Z, et al. Significance of liquid biopsy in glioblastoma—a review. J Biotechnol. 2019;298:82–7. doi: 10.1016/j.jbiotec.2019.04.011 30986516

[29] Welsh JA, Goberdhan DCI, O’Driscoll L, Buzas EI, Blenkiron C, Bussolati B, et al. Minimal information for studies of extracellular vesicles (MISEV2023): from basic to advanced approaches. J Extracell Vesicles. 2024;13(2):e12404. doi: 10.1002/jev2.12404 38326288 PMC10850029

[30] Baranyai T, Herczeg K, Onódi Z, Voszka I, Módos K, Marton N, et al. Isolation of exosomes from blood plasma: qualitative and quantitative comparison of ultracentrifugation and size exclusion chromatography methods. PLoS One. 2015;10(12):e0145686. doi: 10.1371/journal.pone.0145686 26690353 PMC4686892

[31] Koliha N, Wiencek Y, Heider U, Jüngst C, Kladt N, Krauthäuser S, et al. A novel multiplex bead-based platform highlights the diversity of extracellular vesicles. J Extracell Vesicles. 2016;5:29975. doi: 10.3402/jev.v5.29975 26901056 PMC4762227

[32] Wiklander OPB, Bostancioglu RB, Welsh JA, Zickler AM, Murke F, Corso G, et al. Systematic methodological evaluation of a multiplex bead-based flow cytometry assay for detection of extracellular vesicle surface signatures. Front Immunol. 2018;9:1326. doi: 10.3389/fimmu.2018.01326 29951064 PMC6008374

[33] d’Alessandro M, Soccio P, Bergantini L, Cameli P, Scioscia G, Foschino Barbaro MP. Extracellular vesicle surface signatures in IPF patients: a multiplex bead-based flow cytometry approach. Cells. 2021;10(5):1045. 33925174 10.3390/cells10051045 PMC8146446

[34] Ekström K, Crescitelli R, Pétursson HI, Johansson J, Lässer C, Olofsson Bagge R. Characterization of surface markers on extracellular vesicles isolated from lymphatic exudate from patients with breast cancer. BMC Cancer. 2022;22(1):50. doi: 10.1186/s12885-021-08870-w 35012489 PMC8744234

[35] Li L, Görgens A, Mussack V, Pepeldjiyska E, Hartz AS, Rank A, et al. Description and optimization of a multiplex bead-based flow cytometry method (MBFCM) to characterize extracellular vesicles in serum samples from patients with hematological malignancies. Cancer Gene Ther. 2022;29(11):1600–15. doi: 10.1038/s41417-022-00466-1 35477770 PMC9663305

[36] Vacchi E, Burrello J, Di Silvestre D, Burrello A, Bolis S, Mauri P, et al. Immune profiling of plasma-derived extracellular vesicles identifies Parkinson disease. Neurol Neuroimmunol Neuroinflamm. 2020;7(6):e866. doi: 10.1212/NXI.0000000000000866 32817412 PMC7428368

[37] Brahmer A, Geiß C, Lygeraki A, Neuberger E, Tzaridis T, Nguyen TT, et al. Assessment of technical and clinical utility of a bead-based flow cytometry platform for multiparametric phenotyping of CNS-derived extracellular vesicles. Cell Commun Signal. 2023;21(1):276. doi: 10.1186/s12964-023-01308-9 37803478 PMC10559539

[38] Tzaridis T, Weller J, Bachurski D, Shakeri F, Schaub C, Hau P, et al. A novel serum extracellular vesicle protein signature to monitor glioblastoma tumor progression. Int J Cancer. 2023;152(2):308–19. doi: 10.1002/ijc.34261 36054558 PMC13200606

[39] Franceschi S, Lessi F, Morelli M, Menicagli M, Aretini P, Gambacciani C, et al. Exploring extracellular vesicle surface protein markers produced by glioblastoma tumors: a characterization study using in vitro 3d patient-derived cultures. Cancers (Basel). 2024;16(22):3748. doi: 10.3390/cancers16223748 39594703 PMC11592176

[40] Caponnetto F, Dalla E, Mangoni D, Piazza S, Radovic S, Ius T, et al. The miRNA content of exosomes released from the glioma microenvironment can affect malignant progression. Biomedicines. 2020;8(12):564. doi: 10.3390/biomedicines8120564 33287106 PMC7761654

[41] Lynch D, Powter B, Po J, Cooper A, Garrett C, Koh E. Isolation of circulating tumor cells from glioblastoma patients by direct immunomagnetic targeting. Applied Sciences. 2020;10(9):3338. doi: 10.3390/app10093338

[42] Ampofo E, Schmitt BM, Menger MD, Laschke MW. The regulatory mechanisms of NG2/CSPG4 expression. Cell Mol Biol Lett. 2017;22 4. doi: 10.1186/s11658-017-0035-3 28536635 PMC5415841

[43] Erfurt C, Müller E, Emmerling S, Klotz C, Hertl M, Schuler G, et al. Melanoma-associated chondroitin sulphate proteoglycan as a new target antigen for CD4+ T cells in melanoma patients. Int J Cancer. 2009;124(10):2341–6. doi: 10.1002/ijc.24235 19173283

[44] Ilieva K, Cheung A, Mele S, Chiaruttini G, Crescioli S, Griffin M. Chondroitin sulfate proteoglycan 4 and its potential as an antibody immunotherapy target across different tumor types. Front Immun. 2018;8:1911. doi: 10.3389/fimmu.2018.01911 PMC5767725 29375561

[45] Chen X, Ma W-Y, Xu S-C, Liang Y, Fu Y-B, Pang B, et al. The overexpression of epithelial cell adhesion molecule (EpCAM) in glioma. J Neurooncol. 2014;119(1):39–47. doi: 10.1007/s11060-014-1459-5 24906438

[46] Liu Y, Wang Y, Sun S, Chen Z, Xiang S, Ding Z, et al. Understanding the versatile roles and applications of EpCAM in cancers: from bench to bedside. Exp Hemat Oncol. 2022;11(1):97. 10.1186/s40164-022-00352-4 PMC9650829 36369033

[47] Jeon H-M, Kim J-Y, Cho HJ, Lee WJ, Nguyen D, Kim SS, et al. Tissue factor is a critical regulator of radiation therapy-induced glioblastoma remodeling. Cancer Cell. 2023;41(8):1480–1497.e9. doi: 10.1016/j.ccell.2023.06.007 37451272 PMC10530238

[48] Afshar Moghaddam N, Mahsuni P, Taheri D. Evaluation of endoglin as an angiogenesis marker in Glioblastoma. Iran J Pathol. 2015;10(2):89–96. 26351468 PMC4539765

[49] Maddison K, Bowden NA, Graves MC, Tooney PA. Characteristics of vasculogenic mimicry and tumour to endothelial transdifferentiation in human glioblastoma: a systematic review. BMC Cancer. 2023;23(1):185. doi: 10.1186/s12885-023-10659-y 36823554 PMC9948311

[50] Shaifer CA, Huang J, Lin PC. Glioblastoma cells incorporate into tumor vasculature and contribute to vascular radioresistance. Int J Cancer. 2010;127(9):2063–75. doi: 10.1002/ijc.25249 20162571 PMC2932815

[51] Li C, Meng X, Wang L, Ren S, Matei N, Wu G. Mitigating the effects of Endothelin-1 following a minimally invasive surgery reduces the blood-brain barrier permeability in a rabbit model of intracerebral hemorrhage. Brain Hemorrhages. 2022;3(4):177–83. doi: 10.1016/j.hest.2022.06.004

[52] Lee U, Cho E-Y, Jho E-H. Regulation of hippo signaling by metabolic pathways in cancer. Biochim Biophys Acta Mol Cell Res. 2022;1869(4):119201. doi: 10.1016/j.bbamcr.2021.119201 35026349

[53] Skandalis SS. CD44 Intracellular domain: a long tale of a short tail. Cancers (Basel). 2023;15(20):5041. doi: 10.3390/cancers15205041 37894408 PMC10605500

[54] Yu S, Cai X, Wu C, Wu L, Wang Y, Liu Y. Adhesion glycoprotein CD44 functions as an upstream regulator of a network connecting ERK, AKT and Hippo-YAP pathways in cancer progression. Oncotarget. 2015;6(5):2951–65. 25605020 10.18632/oncotarget.3095 PMC4413630

[55] Pietras A, Katz AM, Ekström EJ, Wee B, Halliday JJ, Pitter KL, et al. Osteopontin-CD44 signaling in the glioma perivascular niche enhances cancer stem cell phenotypes and promotes aggressive tumor growth. Cell Stem Cell. 2014;14(3):357–69. doi: 10.1016/j.stem.2014.01.005 24607407 PMC3999042

[56] Lottaz C, Beier D, Meyer K, Kumar P, Hermann A, Schwarz J. Transcriptional profiles of CD133+ and CD133− glioblastoma-derived cancer stem cell lines suggest different cells of origin. Cancer Res. 2010;70(5):2030–40. 20145155 10.1158/0008-5472.CAN-09-1707

[57] Brown DV, Filiz G, Daniel PM, Hollande F, Dworkin S, Amiridis S, et al. Expression of CD133 and CD44 in glioblastoma stem cells correlates with cell proliferation, phenotype stability and intra-tumor heterogeneity. PLoS One. 2017;12(2):e0172791. doi: 10.1371/journal.pone.0172791 28241049 PMC5328356

[58] Sigal DS, Hermel DJ, Hsu P, Pearce T. The role of Globo H and SSEA-4 in the development and progression of cancer, and their potential as therapeutic targets. Future Oncol. 2022;18(1):117–34. doi: 10.2217/fon-2021-1110 34734786

[59] Sivasubramaniyan K, Harichandan A, Schilbach K, Mack AF, Bedke J, Stenzl A, et al. Expression of stage-specific embryonic antigen-4 (SSEA-4) defines spontaneous loss of epithelial phenotype in human solid tumor cells. Glycobiology. 2015;25(8):902–17. doi: 10.1093/glycob/cwv032 25978997 PMC4565992

[60] Lou Y-W, Wang P-Y, Yeh S-C, Chuang P-K, Li S-T, Wu C-Y, et al. Stage-specific embryonic antigen-4 as a potential therapeutic target in glioblastoma multiforme and other cancers. Proc Natl Acad Sci U S A. 2014;111(7):2482–7. doi: 10.1073/pnas.1400283111 24550271 PMC3932869

[61] Yawata T, Higashi Y, Kawanishi Y, Nakajo T, Fukui N, Fukuda H, et al. CD146 is highly expressed in glioma stem cells and acts as a cell cycle regulator. J Neurooncol. 2019;144(1):21–32. doi: 10.1007/s11060-019-03200-4 31147892

[62] Liang Y, Voshart D, Paridaen JTML, Oosterhof N, Liang D, Thiruvalluvan A, et al. CD146 increases stemness and aggressiveness in glioblastoma and activates YAP signaling. Cell Mol Life Sci. 2022;79(8):398. doi: 10.1007/s00018-022-04420-0 35790583 PMC9256581

[63] Jung E-H, Lee H-N, Han G-Y, Kim M-J, Kim C-W. Targeting ROR1 inhibits the self-renewal and invasive ability of glioblastoma stem cells. Cell Biochem Funct. 2016;34(3):149–57. doi: 10.1002/cbf.3172 26923195

[64] Ishikawa T, Ogura Y, Tanaka K, Nagashima H, Sasayama T, Endo M, et al. Ror1 is expressed inducibly by Notch and hypoxia signaling and regulates stem cell-like property of glioblastoma cells. Cancer Sci. 2023;114(2):561–73. doi: 10.1111/cas.15630 36314076 PMC9899608

[65] Latour M, Her NG, Kesari S, Nurmemmedov E. WNT Signaling as a Therapeutic Target for Glioblastoma. Int J Mol Sci. 2021 Aug 5;22(16):8428. 34445128 10.3390/ijms22168428 PMC8395085

[66] Crane CA, Ahn BJ, Han SJ, Parsa AT. Soluble factors secreted by glioblastoma cell lines facilitate recruitment, survival, and expansion of regulatory T cells: implications for immunotherapy. Neuro Oncol. 2012;14(5):584–95. doi: 10.1093/neuonc/nos014 22406925 PMC3337302

[67] DiDomenico J, Lamano JB, Oyon D, Li Y, Veliceasa D, Kaur G, et al. The immune checkpoint protein PD-L1 induces and maintains regulatory T cells in glioblastoma. Oncoimmunology. 2018;7(7):e1448329. doi: 10.1080/2162402X.2018.1448329 29900065 PMC5993506

[68] Lamano JB, Lamano JB, Li YD, DiDomenico JD, Choy W, Veliceasa D, et al. Glioblastoma-derived il6 induces immunosuppressive peripheral myeloid cell PD-L1 and promotes tumor growth. Clin Cancer Res. 2019;25(12):3643–57. doi: 10.1158/1078-0432.CCR-18-2402 30824583 PMC6571046

[69] Ravi VM, Neidert N, Will P, Joseph K, Maier JP, Kückelhaus J, et al. T-cell dysfunction in the glioblastoma microenvironment is mediated by myeloid cells releasing interleukin-10. Nat Commun. 2022;13(1):925. doi: 10.1038/s41467-022-28523-1 35177622 PMC8854421

[70] Himes B, Geiger P, Ayasoufi K, Bhargav A, Brown D, Parney I. Immunosuppression in glioblastoma: current understanding and therapeutic implications. Front Oncol. 2021;11 770561. doi: 10.3389/fonc.2021.770561 34778089 PMC8581618

[71] Anand N, Peh KH, Kolesar JM. Macrophage repolarization as a therapeutic strategy for osteosarcoma. Int J Mol Sci. 2023;24(3):2858. doi: 10.3390/ijms24032858 36769180 PMC9917837

[72] Beier CP, Kumar P, Meyer K, Leukel P, Bruttel V, Aschenbrenner I, et al. The cancer stem cell subtype determines immune infiltration of glioblastoma. Stem Cells Develop. 2012;21(15):2753–61. 10.1089/scd.2011.0660 PMC3464079 22676416

[73] Ellert-Miklaszewska A, Poleszak K, Pasierbinska M, Kaminska B. Integrin signaling in glioma pathogenesis: from biology to therapy. Int J Mol Sci. 2020;21(3):888. 32019108 10.3390/ijms21030888 PMC7037280

[74] Gourlay J, Morokoff AP, Luwor RB, Zhu H-J, Kaye AH, Stylli SS. The emergent role of exosomes in glioma. J Clin Neurosci. 2017;35 13–23. doi: 10.1016/j.jocn.2016.09.021 27771233

[75] Mallawaaratchy DM, Hallal S, Russell B, Ly L, Ebrahimkhani S, Wei H, et al. Comprehensive proteome profiling of glioblastoma-derived extracellular vesicles identifies markers for more aggressive disease. J Neurooncol. 2017;131(2):233–44. doi: 10.1007/s11060-016-2298-3 27770278 PMC5306193

[76] Paolillo M, Schinelli S. Integrins and exosomes, a dangerous liaison in cancer progression. Cancers. 2017;9(8):95. 28933725 10.3390/cancers9080095 PMC5575598

[77] Lane R, Simon T, Vintu M, Solkin B, Koch B, Stewart N, et al. Cell-derived extracellular vesicles can be used as a biomarker reservoir for glioblastoma tumor subtyping. Commun Biol. 2019;2 315. doi: 10.1038/s42003-019-0560-x 31453379 PMC6700082

[78] Spitzberg JD, Ferguson S, Yang KS, Peterson HM, Carlson JCT, Weissleder R. Multiplexed analysis of EV reveals specific biomarker composition with diagnostic impact. Nat Commun. 2023;14(1):1239. doi: 10.1038/s41467-023-36932-z 36870999 PMC9985597

[79] Chonan M, Saito R, Shoji T, Shibahara I, Kanamori M, Sonoda Y, et al. CD40/CD40L expression correlates with the survival of patients with glioblastomas and an augmentation in CD40 signaling enhances the efficacy of vaccinations against glioma models. Neuro Oncol. 2015;17(11):1453–62. doi: 10.1093/neuonc/nov090 26008605 PMC4648302

[80] Yang F, He Z, Duan H, Zhang D, Li J, Yang H. Synergistic immunotherapy of glioblastoma by dual targeting of IL-6 and CD40. Nat Commun. 2021;12(1):3424. doi: 10.1038/s41467-021-33745-5 34103524 PMC8187342

[81] Campanella R, Guarnaccia L, Cordiglieri C, Trombetta E, Caroli M, Carrabba G, et al. Tumor-educated platelets and angiogenesis in glioblastoma: another brick in the wall for novel prognostic and targetable biomarkers, changing the vision from a localized tumor to a systemic pathology. Cells. 2020 Jan 25;9(2):294. 31991805 10.3390/cells9020294 PMC7072723

[82] Ding S, Dong X, Song X. Tumor educated platelet: the novel BioSource for cancer detection. Cancer Cell Int. 2023;23(1):91. doi: 10.1186/s12935-023-02927-5 37170255 PMC10176761

